# Bioorthogonal catalysis for antimicrobial therapy

**DOI:** 10.1097/mm9.0000000000000001

**Published:** 2024-05-13

**Authors:** Aarohi Gupta, William Ndugire, Liang Liu, Soham Chakraborty, Maged Abdelaziz, Derek Rainboth, Vincent M. Rotello

**Affiliations:** 1 Department of Chemistry, University of Massachusetts Amherst, Amherst, Massachusetts, USA.

## 1. Introduction

**Figure 1.**
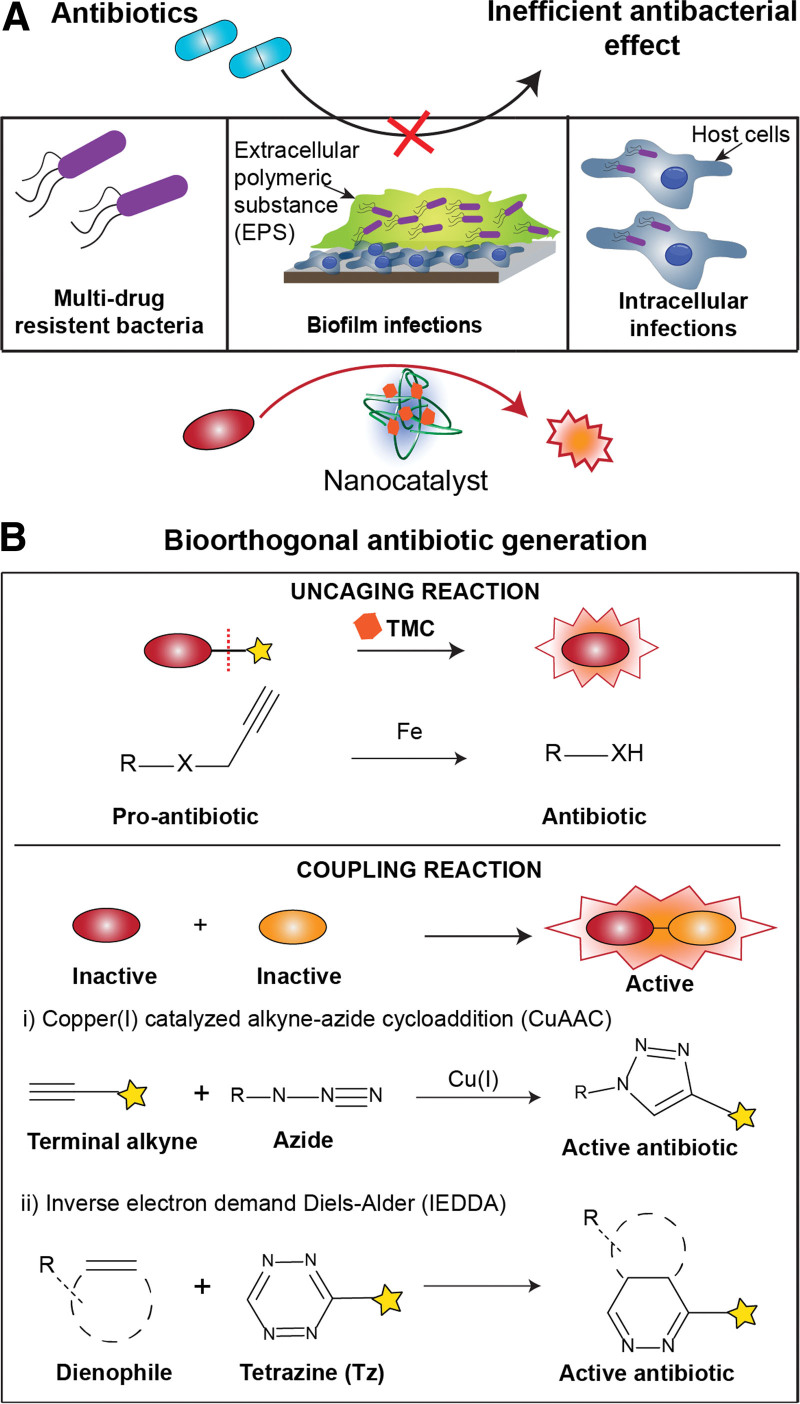
Bioorthogonal antibiotic generation as an alternative to antimicrobial therapy. (A) Traditional antibiotics are inefficient for challenging bacterial infections. (B) Bioorthogonal strategies for in situ antibiotic generation.

Bioorthogonal chemistry uses abiotic reactions that do not interfere with natural biological processes.^[1]^ The use of catalysts in bioorthogonal chemistry offers the ability to control nonnative reactions inside complex living systems, enabling localized generation of bioactive molecules. This emerging field of bioorthogonal catalysis has been integrated into a wide range of synthetic biochemical reactions, including prodrug activation, protein transformation, and cellular engineering. In antibacterial applications, bioorthogonal catalysis enables the positioning of these catalytic reactions within close proximity of microbes to allow continual production of antimicrobials at high local concentrations. This strategy is both effective and timely in addressing increasingly challenging infections such as bacterial biofilms and intracellular pathogens (Figure 1).^[1]^ Traditional antibiotics fare poorly in these therapies as they often fail to penetrate biofilms and cell membranes at sufficient concentrations necessary to kill microbes.^[2,3]^ The alarming spread of antimicrobial resistance to antibiotics has also significantly reduced their efficacy toward recalcitrant infections.^[4]^ Additionally, a lack of novel antibiotics in the discovery pipeline worsens the ongoing crisis. Consequently, recent developments in bioorthogonal catalytic activation of antibiotic prodrugs provide a potential solution to both resistance development and access to bacteria (Figure 1). These catalytic reactions also offer chemical tools for utilizing existing antibiotics more effectively by increasing their production at infection sites.

## 2. Discussion

Bioorthogonal reactions applied in antimicrobial therapy can be broadly categorized into uncaging reactions and coupling reactions (Figure 1).^[1]^ Uncaging reactions involve the removal of a cleavable group by a transition metal catalyst (TMC) (Figure 1B). A protecting group, such as aryl azide, conjugated to antibiotics results in pro-antibiotics that are stable and biologically inert to minimize side effects.^[4–6]^ The caged molecule can then be reactivated by TMC-mediated cleavage at the site of infection. However, the direct use of TMCs in uncaging reactions is challenging as they tend to be hydrophobic and are deactivated in physiological media.^[1]^ Encapsulation of TMCs within nanomaterial scaffolds separates the catalyst from the biological environment limiting these effects. As the interface between the catalyst and the biological systems, the nanomaterial platform provides a tool for tuning these interactions to maximize antibiotic efficacy.

**Figure 2.**
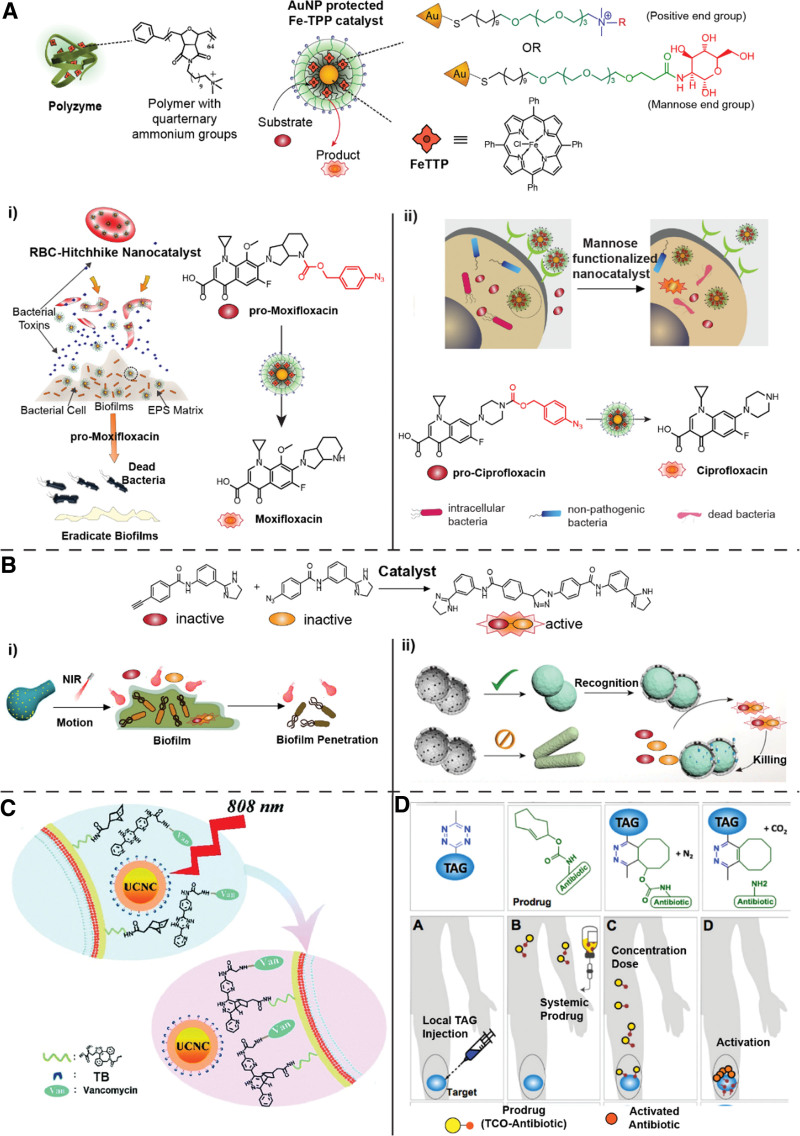
Bioorthogonal approaches to treat challenging infections. (A) Use of either functionalized polymers or gold nanoparticles for protected transition metal catalyst-based antibiotic generation. (i) Selective targeting of biofilm infections with nanocatalysts hitchhiked on red blood cells. Adapted from Gupta et al.^[6]^ with permission from the Royal Society of Chemistry. (ii) Eradication of intracellular infections in macrophages through targeting of CD206 receptors with mannose-presenting nanocatalysts. Adapted from Hardie et al.^[7]^ with permission from the Royal Society of Chemistry. (B) CuAAC-based coupling of alkyne-azide precursors. (i) Near-IR activation of molecular motors embedded with Cu catalysts for biofilm penetration and CuAAC activity. Adapted with permission from Liu et al.^[8]^ Copyright 2022 American Chemical Society. (ii) Bacteria morphology selective bioorthogonal catalysts for strain-selective killing through CuAAC activation. Adapted with permission from Niu et al.^[9]^ Copyright 2021 American Chemical Society. (C) Near-IR-controlled conjugation of vancomycin to methicillin-resistant *S aureus* peptidoglycans through inverse electron-demand Diels−Alder using Nd^3+^ upconversion nanocrystals. Reproduced from Du et al.^[10]^ with permission from the Royal Society of Chemistry. (D) Tetrazine-modified alginate gel for in situ activation of trans-cyclooctene-modified vancomycin and daptomycin prodrugs. Adapted from Czuban et al.^[11]^ under the terms of the CC-BY Creative Commons Attribution 4.0 International License (https://creativecommons.org/licenses/by/4.0/).

A common strategy in targeting bacterial biofilms with TMC-encapsulating platforms is to design nanomaterials with positive charges to enhance penetration.^[5]^ Bacterial biofilms represent crucial targets for bioorthogonal therapeutics, as their development leads to persistent infections on wounds and medical devices. A major hurdle in treating biofilms is the impenetrable extracellular polymeric substance layer that shields the bacterial cells within and prevents the entry of antimicrobial agents. Lipopolysaccharides and excretion of genetic material confer an overall negative charge to the surface of biofilms. Gupta et al. utilized a quaternary ammonium polymer with hydrophobic side chains as carriers for 5,10,15,20-tetraphenyl-21H,23H-porphine (TPP)-containing complex, [Fe(TPP)]Cl for better penetration into *Escherichia coli* or *Pseudomonas aeruginosa* biofilms (Figure 2A). The TMCs were readily stabilized by the hydrophobic environment and the cationic charge facilitated the penetration into biofilms. These polymer nanocatalysts (polyzymes) were utilized to uncage aryl-azide carbamate-protected moxifloxacin or ciprofloxacin within biofilms showing efficient drug activation. The polyzymes also demonstrated excellent biocompatibility, showing minimal toxicity against NIH/3T3 fibroblast cells. This selectivity toward pathogens relative to host is essential for the use of this strategy in the clinic.

Another strategy for biofilm-selective bioorthogonal accumulation of antibiotics was designed by Gupta et al.^[6]^ using red blood cells (RBCs) as the delivery vehicle for Fe catalysts (Figure 2Ai). RBCs provide a natural mode of targeted cargo release at biofilms because they are hemolyzed by bacterial toxins, which are abundant in bacterial biofilms. Additionally, RBCs are biocompatible, possess low immunogenicity and long circulation times in vivo. To load the catalysts onto RBCs, [Fe(TPP)]Cl was first encapsulated within the monolayer of positively charged ~2 nm gold nanoparticles (AuNPs), which were then attached onto negatively charged erythrocytes. The RBC nanocatalysts could selectively activate pro-moxifloxacin to successfully eradicate *E coli* and *Staphylococcus aureus* biofilms over nonvirulent bacterial strains and macrophages. The hitchhiked [FeTPP]Cl on RBCs were nonimmunogenic as measured by tumor necrosis factor alpha cytokine expression in RAW 264.7 macrophage cells, an important consideration for nanoparticles (NPs) in nanomedicine. Another area where traditional antibiotics are ineffective is in the treatment of intracellular infections.^[2]^ The challenge arises from their poor penetration into host cell membranes and susceptibility to enzymatic degradation in the cytosol. Certain bacteria such as *Salmonella* exploit these flaws to hide from antibiotics and immune cells by dwelling within phagocytes, for example, macrophages. To tackle this problem with bioorthogonal catalysis, Hardie et al.^[7]^ reported a mannose-targeting strategy directed toward macrophages infected with *Salmonella* (Figure 2Aii). [Fe(TPP)]Cl catalysts were encapsulated in AuNPs functionalized with d-mannose ligands. These nanocatalysts were selectively uptaken by macrophages through interaction with mannose-binding CD206 receptors. By this enhanced accumulation, the internalized nanocatalysts allowed sufficient generation of ciprofloxacin in macrophages that killed the intracellular bacteria. The local generation of the broad-spectrum ciprofloxacin within macrophages was not toxic to beneficial gut bacteria such as *Lactobacillus*, unlike the direct application of the antibiotic, demonstrating the capability of targeted bioorthogonal catalysts as drug factories, minimizing off-target effects of therapeutics.

The other bioorthogonal antimicrobial approach utilizes coupling reactions where 2 nontoxic substrates react to generate antimicrobial activity (Figure 1B). The classic click coupling reaction Cu-catalyzed azide-alkyne cycloaddition (CuAAC) was used by Niu et al.^[8,9]^ to construct a 1,4-triazole antimicrobial in situ from inactive azide and alkyne precursors (Figure 2B). Based on this chemistry, they proceeded to tackle 2 hurdles that face antibacterial therapy: (1) biofilm penetration (Figure 2Bi) and (2) strain selectivity in bacteria-killing (Figure 2Bii). To penetrate biofilm and perform CuAAC, they fabricated carbonaceous calabash-shaped nanomaterials implanted with copper (CNC-Cu) that were capable of molecular motion.^[8]^ CNC-Cu have a gourd-like morphology that permits forward locomotion as heated water is expelled from their cavity under near-IR (NIR) irradiation. With continual motion, the CNC-Cu can be propelled into biofilms from where local generation of antibiotics can be achieved. The nanocatalysts were successfully used to clear *E coli* biofilms in a mouse implant-related periprosthetic infection model. The successful wound healing in an in vivo murine model demonstrates the translatability of TMC-mediated bioorthogonal catalysis in biofilm treatment.

To achieve strain-selective antibacterial treatment, Niu et al.^[9]^ developed shape-selective bioorthogonal catalysts that could select between different bacterial strains based on cell morphology. To do this, the group deposited layers of Cu and SiO_2_ onto *S aureus* and *E coli*, then etched away the organic material to recover the empty bacterial-templated shells. These antibody structures could preferentially bind to bacteria of the same shape providing a method for delivery of the Cu catalyst selectively. With Cu-mediated antimicrobial activation, spherical *S aureus* could be selectively killed over rod-shaped *E coli* bacteria and vice versa.

The inverse electron-demand Diels−Alder (IEDDA) reaction between tetrazines and a strained dienophile is a bioorthogonal coupling reaction that does not require a TMC—a potential source of cytotoxicity (Figure 1Bii). Du et al.^[10]^ utilized this reaction to target vancomycin to methicillin-resistant *S aureus* (MRSA) (Figure 2C). Vancomycin is a last resort antibiotic that kills MRSA by complexing with the peptidoglycan of the cell wall leading to cell lysis. However, vancomycin-resistant MRSA strains have restructured cell walls that prevent binding with the antibiotic. Du and coworkers modified MRSA peptidoglycans with norbornene to allow IEDDA coupling with tetrazine-derivatized vancomycin (Van-Tz) thereby increasing local antibiotic concentration. To control the reaction, the group developed Van-dHTz, an unreactive precursor that requires oxidization to IEDDA-reactive Van-Tz. This oxidation was achieved using Nd^3+^-based upconversion nanocrystals coated with toluidine blue O (TB-UCNC). When excited by 808 nm NIR light, TB-UCNC catalyzes the oxidation of Van-dHTz to Van-Tz, which can then conjugate with the norbornene-tagged peptidoglycan. Further testing of this strategy with other Gram-positive bacteria (*B subtilis*, *E faecalis*, and *E faecium*) showed a general trend of 6- to 7-fold drop in Minimum inhibitory concentration (MIC) over vancomycin.

Czuban et al.^[11]^ reported the use of a bioorthogonal IEDDA strategy using trans-cyclooctene (TCO) as the strained dienophile (Figure 2D). In their approach, an alginate gel with tetrazine-modification (TAG) was injected at the site of infection, and a TCO-modified vancomycin or daptomycin prodrug was administered intravenously. When the prodrug encounters the TAG, it reacts via IEDDA chemistry and then isomerizes to release the active drug at the infection site. The prodrug approach avoids issues facing the systemic administration of antibiotics especially in treatments of antibiotic-resistant bacterial strains in biofilm-infected implants. In these therapies, high dosages are needed that can cause significant off-target effects, including the destruction of commensal gut bacteria. The TAG activation of TCO-vancomycin could successfully eliminate bioluminescent MRSA, injected into the thighs of neutropenic mice, by 3 orders of magnitude compared to unmodified alginate gel. While IEDDA chemistry is fast and selective, providing a biocompatible approach for generating antimicrobials bioorthogonally, the requirement of 2 substrates and their mutual accessibility should be considered for sufficient antimicrobial production.

## 3. Conclusion

The rising rates of antimicrobial resistance have placed great stress on the traditional antibiotic pipeline. As newer drugs develop resistance, it is imperative to develop strategies that reduce resistance generation and increase the efficiency of current therapies. Bioorthogonal catalysis promises a timely intervention in this vein through controlled activation of inactive prodrugs. A common approach to achieve this selectivity is by localizing the catalyst close to the site of bacterial infection. Nanomaterial scaffolds have proved invaluable in this process, allowing targeting of planktonic bacteria, intracellular infections, and biofilms. Nanomaterials also provide the ability to modulate the catalytic activity of TMCs using exogenous agents such as pH, light, and temperature. As such, the progress of bioorthogonal catalysis in antimicrobial applications goes hand in hand with the advancement of new nanomaterials that will enhance both the stability and specificity of catalysts operating within biological settings.

The toxicity of metal catalysts is a significant challenge that faces the translation of bioorthogonal catalysis to clinical application. However, NP-protected TMCs showed minimal mammalian cytotoxicity and hemolytic activity in all studies, suggesting that TMC toxicity can be reduced using NPs. Another approach to mitigate toxicity is the formulation of TMCs with biodegradable carriers and the use of less toxic TMCs, such as naturally occurring Fe catalysts, for example, hemin. Alternatively, the development of catalyst-free reactions such as IEDDA can obviate the need for TMCs.

There is still a significant knowledge gap between TMC administration and the subsequent fate of catalysts intracellularly and systemically, and their long-term effects, issues that need to be addressed in ongoing research. Nonetheless, as highlighted in this perspective, the bioorthogonal approach to addressing persistent and resistant infections is rapidly progressing. Advanced imaging techniques could be employed to monitor the distribution and activity of these catalysts in real time, offering more comprehensive insights into their behavior within living organisms. Reports of successfully treated infections in vivo that the next step—translating to higher mammals and human trials—is a challenging but achievable goal.

## Conflicts of interests

The authors declare that they have no conflicts of interest.

## Funding

This research was funded by the National Institutes of Health grants EB022641 and AI134770 (Vincent M. Rotello). William Ndugire was funded by EB022641-S.
